# Supplementary material for: Gut Digestive Function and Microbiome after Correction of Experimental Dysbiosis in Rats by Indigenous Bifidobacteria
Source: Microorganisms. 2021 Mar 4;9(3):522. doi: 10.3390/microorganisms9030522 (PMC8001560; doi:10.3390/microorganisms9030522)
Supplement: Supplementary file 1 [file microorganisms-09-00522-s001.zip › Suppl. mater. 03-03-21/Supplementary-Materials-_Tables-S1_S2_S3_-Captions-of-Figures-S1_S2_.docx]

**Table S1.** Multifactor analysis of metagenome differences between groups Bf and C1.

Multivariate linear model

============================================================

------------------------------------------------------------

Intercept Value Num DF Den DF F Value Pr > F

------------------------------------------------------------

Wilks' lambda 0.0528 7.0000 6.0000 15.3825 0.0019

Pillai's trace 0.9472 7.0000 6.0000 15.3825 0.0019

Hotelling-Lawley trace 17.9462 7.0000 6.0000 15.3825 0.0019

Roy's greatest root 17.9462 7.0000 6.0000 15.3825 0.0019

------------------------------------------------------------

------------------------------------------------------------

Condition Value Num DF Den DF F Value Pr > F

------------------------------------------------------------

Wilks' lambda 0.1226 7.0000 6.0000 6.1346 0.0209

Pillai's trace 0.8774 7.0000 6.0000 6.1346 0.0209

Hotelling-Lawley trace 7.1571 7.0000 6.0000 6.1346 0.0209

Roy's greatest root 7.1571 7.0000 6.0000 6.1346 0.0209

============================================================

MANOVA analyses in Python stats model.

**Table S2.** Multifactor analysis of metagenome differences between groups C1 and C0.

-----------------------------------------------------------

Multivariate linear model

===========================================================

-----------------------------------------------------------

Intercept Value Num DF Den DF F Value Pr > F

-----------------------------------------------------------

Wilks' lambda 0.0988 7.0000 8.0000 10.4298 0.0018

Pillai's trace 0.9012 7.0000 8.0000 10.4298 0.0018

Hotelling-Lawley trace 9.1261 7.0000 8.0000 10.4298 0.0018

Roy's greatest root 9.1261 7.0000 8.0000 10.4298 0.0018

-----------------------------------------------------------

-----------------------------------------------------------

Condition Value Num DF Den DF F Value Pr > F

-----------------------------------------------------------

Wilks' lambda 0.2076 7.0000 8.0000 4.3626 0.0278

Pillai's trace 0.7924 7.0000 8.0000 4.3626 0.0278

Hotelling-Lawley trace 3.8173 7.0000 8.0000 4.3626 0.0278

Roy's greatest root 3.8173 7.0000 8.0000 4.3626 0.0278

===========================================================

MANOVA analyses in Python stats model.

**Table S3.** Multifactor analysis of metagenome differences between groups Bf and C0.

Multivariate linear model

============================================================

------------------------------------------------------------

Intercept Value Num DF Den DF F Value Pr > F

------------------------------------------------------------

Wilks' lambda 0.0821 7.0000 8.0000 12.7836 0.0009

Pillai's trace 0.9179 7.0000 8.0000 12.7836 0.0009

Hotelling-Lawley trace 11.1857 7.0000 8.0000 12.7836 0.0009

Roy's greatest root 11.1857 7.0000 8.0000 12.7836 0.0009

------------------------------------------------------------

------------------------------------------------------------

Condition Value Num DF Den DF F Value Pr > F

------------------------------------------------------------

Wilks' lambda 0.3571 7.0000 8.0000 2.0571 0.1667

Pillai's trace 0.6429 7.0000 8.0000 2.0571 0.1667

Hotelling-Lawley trace 1.8000 7.0000 8.0000 2.0571 0.1667

Roy's greatest root 1.8000 7.0000 8.0000 2.0571 0.1667

============================================================

MANOVA analyses in Python stats model.

**Figure captions**

1. Figure S1. Alpha-diversity indices of fecal samples metagenome of rats from different groups.

2. Figure S2. Results of correlation analysis between statically important bacterial genera in intestinal microbiome of rats from different groups (9^th^ day of experiments).

Data are represented in a heatmap form using a Python seaborn library.
