# Supplementary figures and images for: Gut Digestive Function and Microbiome after Correction of Experimental Dysbiosis in Rats by Indigenous Bifidobacteria
Source: Microorganisms. 2021 Mar 4;9(3):522. doi: 10.3390/microorganisms9030522 (PMC8001560; doi:10.3390/microorganisms9030522)

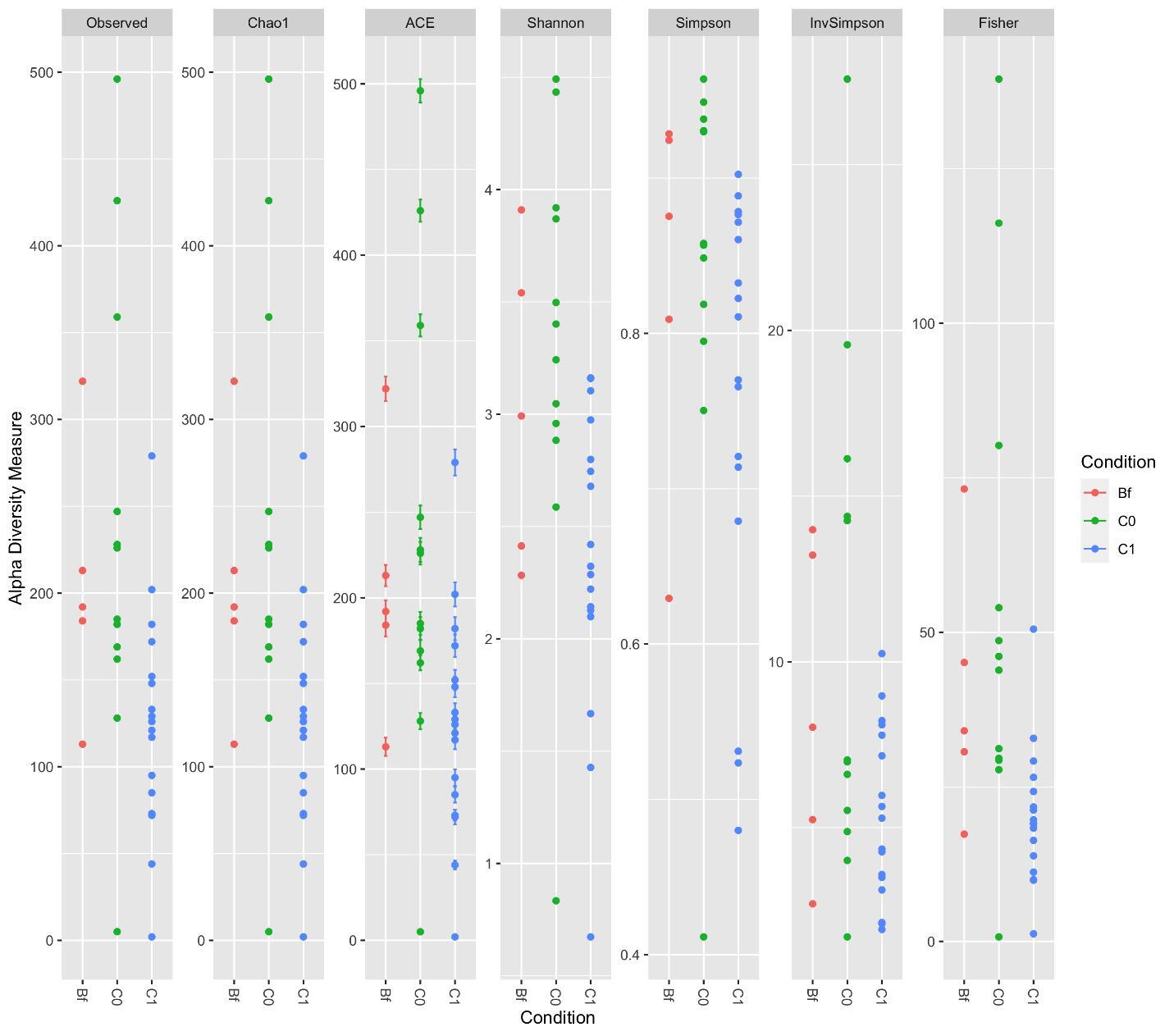

Supplement: Supplementary file 1 [file microorganisms-09-00522-s001.zip › Suppl. mater. 03-03-21/Figure-S1-_Supplementary-Materials_.jpeg]

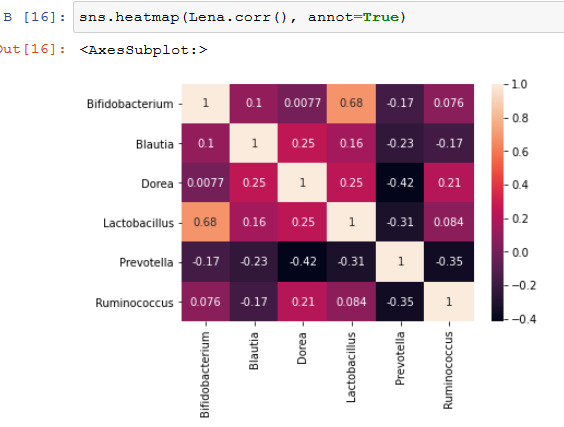

Supplement: Supplementary file 1 [file microorganisms-09-00522-s001.zip › Suppl. mater. 03-03-21/Figure-S2-_Supplementary-Materials_.jpeg]
