# Supplementary material for: Gut Digestive Function and Microbiome after Correction of Experimental Dysbiosis in Rats by Indigenous Bifidobacteria
Source: Microorganisms. 2021 Mar 4;9(3):522. doi: 10.3390/microorganisms9030522 (PMC8001560; doi:10.3390/microorganisms9030522)
Supplement: Supplementary file 1 [file microorganisms-09-00522-s001.zip › Suppl. mater. 03-03-21/Conditions-of-sequencing-_Supplem.-Materials_.doc]

| Conditions | |
| --- | --- |
| **IEM36** | C1 |
| **IEM37** | C1 |
| **IEM38** | C1 |
| **IEM39** | C1 |
| **IEM45** | C0 |
| **IEM46** | C1 |
| **IEM47** | C1 |
| **IEM48** | C0 |
| **IEM49** | C0 |
| **IEM53** | Bf |
| **IEM65** | Bf |
| **IEM85** | Bf |
| **IEM97** | C0 |
| **IEM98** | C0 |
| **IEM102** | C0 |
| **IEM105** | Bf |
| **IEM106** | Bf |
| **IEM113** | C0 |
| **IEM119** | C1 |
| **IEM120** | C1 |
| **IEM121** | C1 |
| **IEM122** | C1 |
| **IEM123** | C1 |
| **IEM125** | C0 |
| **IEM126** | C0 |
| **IEM127** | C0 |
| **IEM128** | C1 |
| **IEM130** | C0 |
| **IEM131** | C1 |
| **IEM132** | C0 |
| **IEM133** | C1 |
| **IEM135** | C1 |
| **IEM139** | C1 |
| **IEM142** | C1 |
